# Supplementary material for: Identification of the kinase STK25 as an upstream activator of LATS signaling
Source: Nat Commun. 2019 Apr 4;10:1547. doi: 10.1038/s41467-019-09597-w (PMC6449379; doi:10.1038/s41467-019-09597-w)
Supplement: Supplementary file 1 — Supplementary Information [file 41467_2019_9597_MOESM1_ESM.pdf]

Supplementary Information

Identification of STK25 as an upstream activator of LATS signaling.

Lim et al.

This supplementary information section contains the following:

Supplementary figures 1-8

- Supplementary figure 1: pages 2-3
- Supplementary figure 2: pages 4-5
- Supplementary figure 3: page 6
- Supplementary figure 4: page 8
- Supplementary figure 5: page 9
- Supplementary figure 6: page 10
- Supplementary figure 7: page 11
- Supplementary figure 8: pages 12-16

Supplementary tables 1-5

- Supplementary table 1: page 17
- Supplementary table 2: page 18
- Supplementary table 3: page 19
- Supplementary table 4: page 20-21
- Supplementary table 5: pages 22

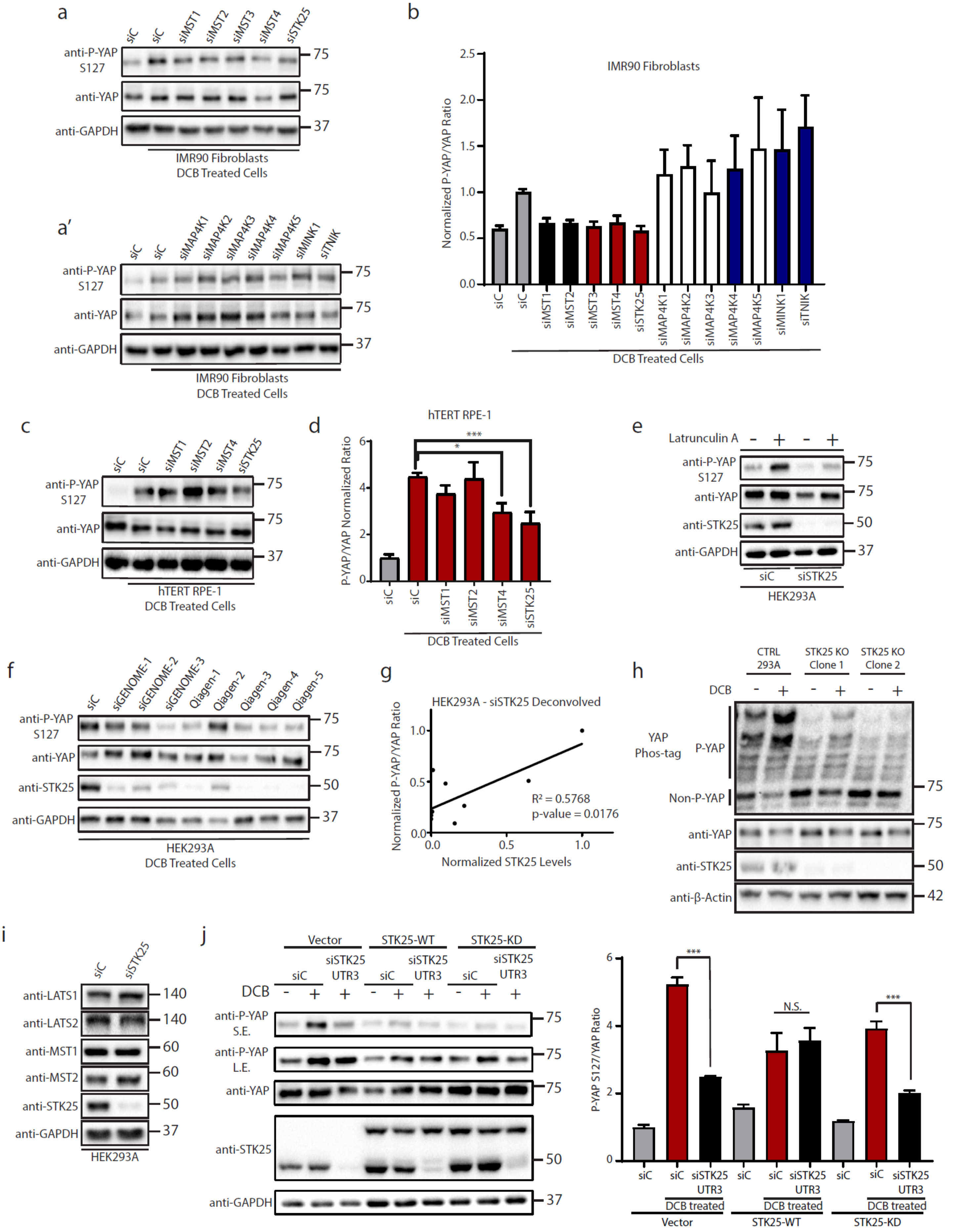
a
siC
siC
siMST1
siMST2
siMST3
siMST4
siSTK25
anti-P-YAP S127
anti-YAP
anti-GAPDH
75
75
37
IMR90 Fibroblasts
DCB Treated Cells
a′
siC
siC
siMAP4K1
siMAP4K2
siMAP4K3
siMAP4K4
siMAP4K5
siMINK1
siTNIK
anti-P-YAP S127
anti-YAP
anti-GAPDH
75
75
37
IMR90 Fibroblasts
DCB Treated Cells
b
IMR90 Fibroblasts
Normalized P-YAP/YAP Ratio
2.5
2.0
1.5
1.0
0.5
0.0
siC
siC
siMST1
siMST2
siMST3
siMST4
siSTK25
siMAP4K1
siMAP4K2
siMAP4K3
siMAP4K4
siMAP4K5
siMINK1
siTNIK
DCB Treated Cells
c
siC
siC
siMST1
siMST2
siMST4
siSTK25
anti-P-YAP S127
anti-YAP
anti-GAPDH
75
75
37
hTERT RPE-1
DCB Treated Cells
d
hTERT RPE-1
P-YAP/YAP Normalized Ratio
6
4
2
0
***
*
siC
siC
siMST1
siMST2
siMST4
siSTK25
DCB Treated Cells
e
Latrunculin A
-
+
-
+
anti-P-YAP S127
anti-YAP
anti-STK25
anti-GAPDH
75
75
50
37
siC
siSTK25
HEK293A
f
siC
siGENOME-1
siGENOME-2
siGENOME-3
Qiagen-1
Qiagen-2
Qiagen-3
Qiagen-4
Qiagen-5
anti-P-YAP S127
anti-YAP
anti-STK25
anti-GAPDH
75
75
50
37
HEK293A
DCB Treated Cells
g
HEK293A - siSTK25 Deconvolved
Normalized P-YAP/YAP Ratio
1.0
0.5
0.0
0.0
0.5
1.0
R² = 0.5768
p-value = 0.0176
Normalized STK25 Levels
h
CTRL 293A
STK25 KO Clone 1
STK25 KO Clone 2
DCB
-
+
-
+
-
+
YAP Phos-tag
P-YAP
Non-P-YAP
75
anti-YAP
75
anti-STK25
50
anti-β-Actin
42
i
siC
siSTK25
anti-LATS1
anti-LATS2
anti-MST1
anti-MST2
anti-STK25
anti-GAPDH
140
140
60
60
50
37
HEK293A
j
Vector
STK25-WT
STK25-KD
siC
siSTK25 UTR3
siC
siSTK25 UTR3
siC
siSTK25 UTR3
DCB
-
+
+
-
+
+
-
+
+
anti-P-YAP S.E.
anti-P-YAP L.E.
anti-YAP
anti-STK25
anti-GAPDH
75
75
75
50
37
P-YAP S127/YAP Ratio
6
4
2
0
***
N.S.
***
siC
siC
siSTK25 UTR3
siC
siC
siSTK25 UTR3
siC
siC
siSTK25 UTR3
DCB treated
DCB treated
DCB treated
Vector
STK25-WT
STK25-KD

Supplementary Figure 1. Loss of STK25 decreases YAP phosphorylation in response to actin disruption. **a.** Representative immunoblot of YAP phosphorylation following treatment with 10 µM DCB in IMR90 fibroblasts transfected with the indicated siRNA. **b.** Quantitation of the IMR90 focused kinome screen (n=3 biological replicates, *p<0.05 by One-Way ANOVA with Dunnett's post-hoc analysis). **c.** Representative immunoblot of YAP phosphorylation following treatment with 10 µM DCB in hTERT-RPE-1 cells transfected with the siRNA. **d.** Quantitation of the RPE-1 secondary kinome screen (n=4 biological replicateds, *p<0.05, ***p<0.001 by One-Way ANOVA with Dunnett's post-hoc analysis). **e.** Representative immunoblot of YAP phosphorylation following treatment with 1 µg/mL Latrunculin A in HEK293A cells transfected with the indicated siRNA. **f.** Immunoblot and **g.** quantification of YAP phosphorylation and STK25 protein levels following treatment with 10 µM DCB in HEK293A cells transfected with the indicated siRNA. STK25 protein levels were normalized to GAPDH protein levels and plotted against the respective P-YAP/YAP ratios. Linear regression was performed to assess whether a correlation existed between levels of STK25 protein expression and P-YAP/YAP ratios (p=0.0176, Pearson's $R^2 = 0.5768$). **h.** Global phosphorylation status of YAP was assessed using phos-tag gel electrophoresis in Control and STK25 KO 293A cells following treatment with 10 µM DCB for one hour; DMSO served as vehicle control. **i.** Lysates collected from HEK293A cells transfected with either control siRNA or siRNAs targeting STK25 were assessed for perturbations in levels of total LATS1, LATS2, MST1, and MST2. **j.** Immunoblot and quantitation of phosphorylated YAP levels following treatment with 10 µM DCB in HEK293A cells stably expressing wild-type STK25 (STK25-WT), kinase-dead STK25 (STK25-KD), or vector control (Vector) transfected with the indicated siRNAs. (n=3 biological replicates; ***p<0.001, N.S.: not significant; One-way ANOVA with Tukey's post-hoc analysis). S.E. stands for Short Exposure; L.E. stands for Long Exposure. All data presented as mean ± SEM unless otherwise noted.

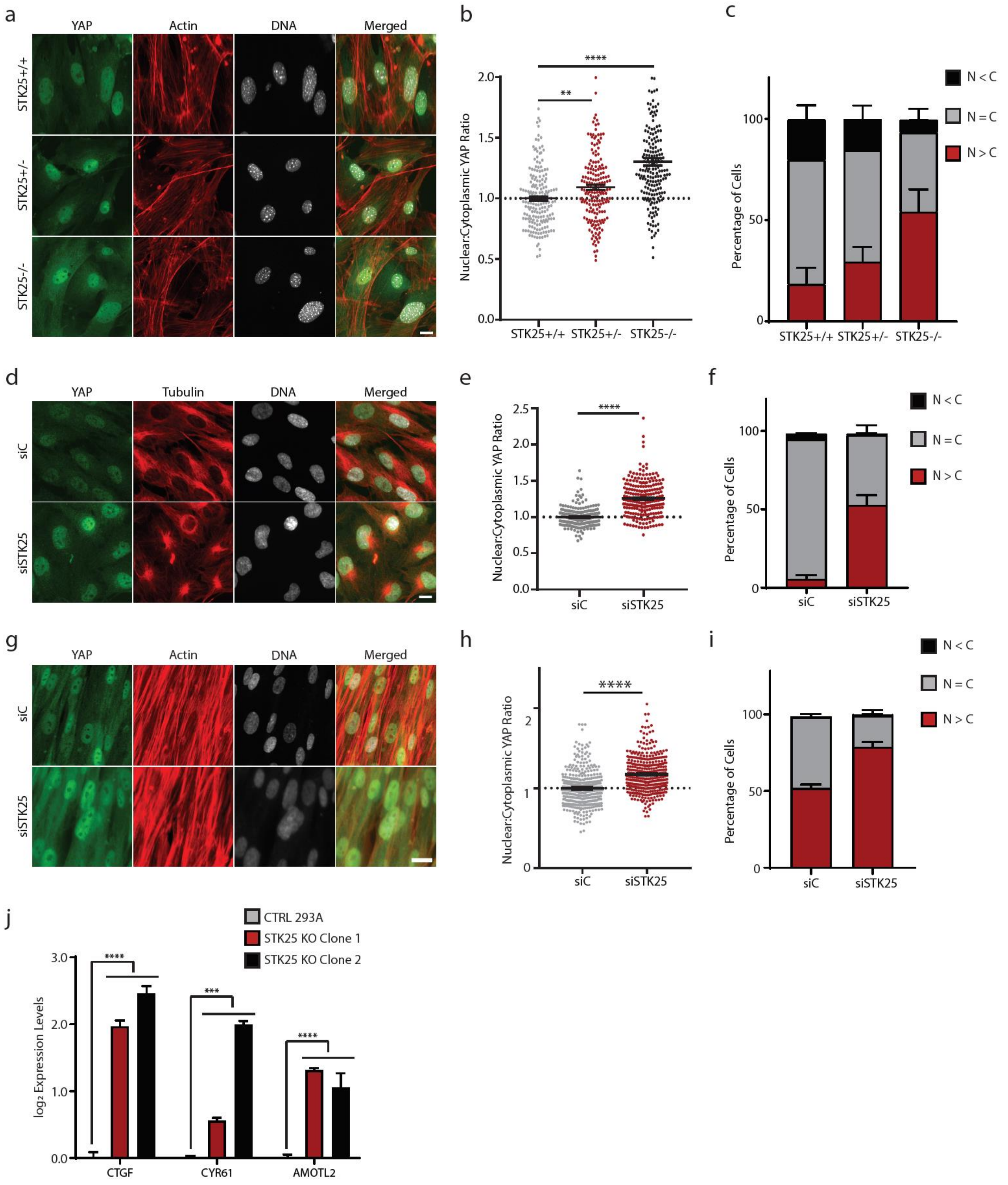

Supplementary Figure 2. Loss of STK25 increases levels of active, nuclear YAP.
a. MEFs isolated from STK25+/+, STK25+/-, and STK25-/- mice were plated on coverslips and stained for YAP (Green), Actin (Red), and DNA (White). Scale bar, 20 µm. **b.** YAP intensity was quantified and nuclear:cytoplasmic ratios were calculated for the MEF images (n>150 per group over 3 biological replicates;

p<0.01, ****p<0.0001 by Kruskal-Wallis test with Dunn's post-test). **c. YAP localization in the MEFs was quantified (n=3 biological replicates, N>C, YAP is enriched in the nucleus; N=C, YAP is evenly distributed between the nucleus and the cytoplasm; N<C, YAP is enriched in the cytoplasm). **d.** hTERT-RPE-1 transfected with the indicated siRNA were stained for YAP (Green), Tubulin (Red), and DNA (White). Scale bar, 20 µm. **e.** YAP intensity was quantified and nuclear:cytoplasmic ratios were calculated for the RPE-1 experiments (n=225 over 3 biological replicates; ****p<0.0001 by Mann-Whitney test). **f.** YAP localization in the hTERT-RPE-1 cells was quantified (n=3 biological replicates, N>C, YAP is enriched in the nucleus; N=C, YAP is evenly distributed between the nucleus and the cytoplasm; N<C, YAP is enriched in the cytoplasm). **g.** IMR90 fibroblasts transfected with the indicated siRNA were stained for YAP (Green), Actin (Red), and DNA (White). Scale bar, 20 µm. **h.** YAP intensity was quantified and nuclear:cytoplasmic ratios were calculated (n=300 per group over 4 biological replicates; ****p<0.0001, Mann-Whitney test). **i.** YAP localization in the IMR90 cells was quantified (n=4 biological replicates, N>C, YAP is enriched in the nucleus; N=C, YAP is evenly distributed between the nucleus and the cytoplasm; N<C, YAP is enriched in the cytoplasm). **j.** qPCR analysis of YAP-target gene expression in control and STK25 KO HEK293A cells (n=4 biological replicates; ***p<0.001, ****p<0.0001, One-way ANOVA with Dunnett's post-hoc analysis). All data presented as mean ± SEM.

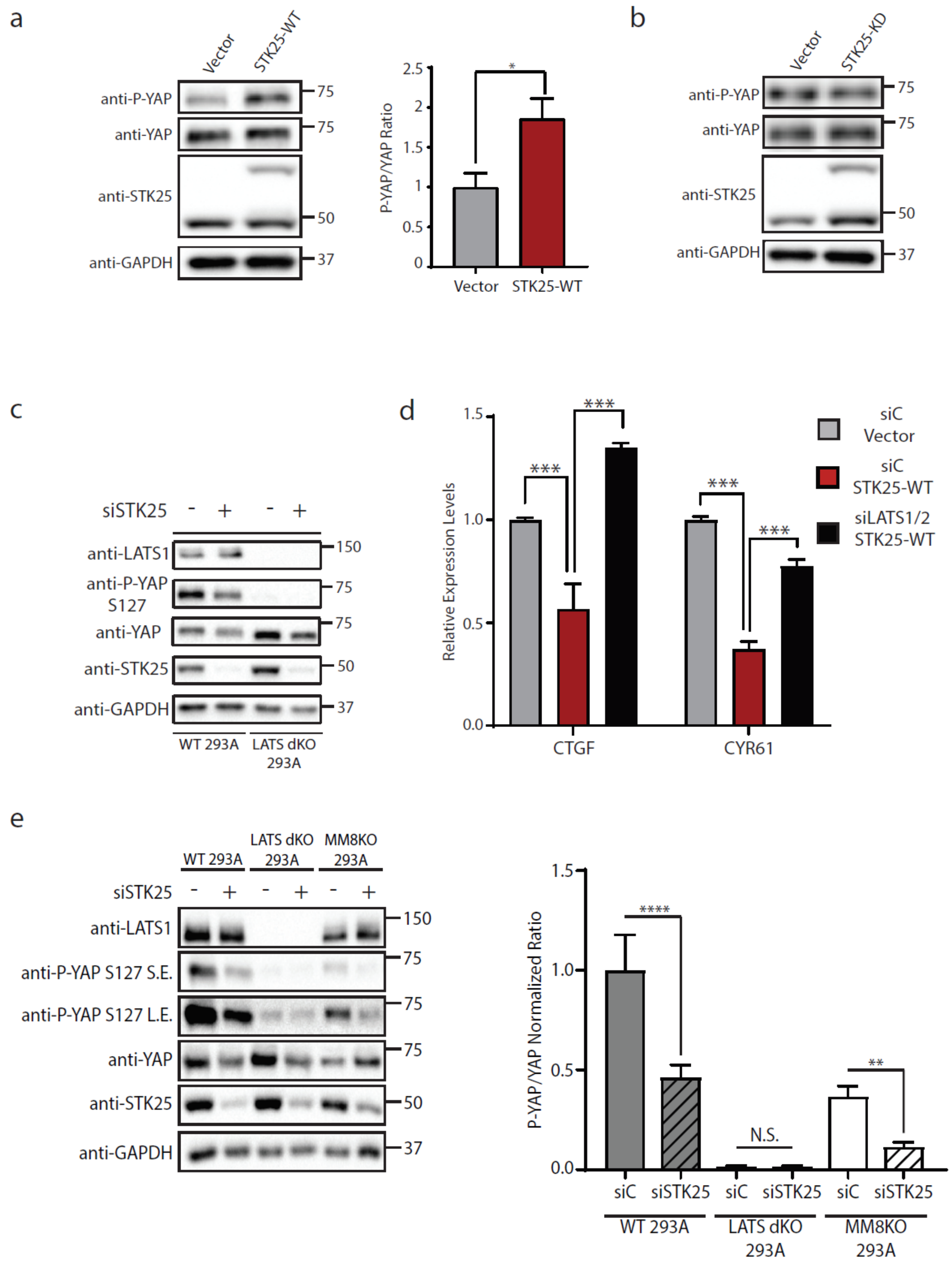

Supplementary Figure 3. STK25 requires LATS1/2 for its inhibitory effects on YAP.
a. Immunoblot and quantitation of phosphorylated YAP levels in HEK293A cells stably expressing wild-type STK25 (STK25-WT) or vector control (Vector) (n=3 biological replicates; *p<0.05, paired t-test). **b.** Immunoblot and quantitation of phosphorylation YAP levels in HEK293A cells stably expressing kinase-dead STK25 (STK25-KD) or vector control (Vector) (n=3 biological replicates; N.S. stands for not significant. paired t-test) **c.** Representative immunoblot of phosphorylated YAP following STK25 knockdown in wild-type and LATS dKO HEK293A. Cells were grown to confluence in order to activate Hippo signaling to induce YAP phosphorylation. **d.** qRT-PCR analysis of YAP-target gene expression in HEK293A cells stably overexpressing either wild-type STK25 (STK25-WT) or vector control (Vector) after transfection with control siRNA or siRNAs targeting LATS1 and LATS2 (n=3 biological replicates; ***p<0.001, One-way ANOVA with Dunnett's post-hoc analysis; DF=2, 6; F=30.08). **e.** Representative immunoblot and quantitation of phosphorylated YAP following STK25 knockdown in wild-type, LATS dKO, and MM8KO HEK293A cells. Cells were grown to confluence in order to activate Hippo signaling and promote YAP phosphorylation. (n=5 biological replicates, ****p<0.0001, **p<0.01, N.S.: not significant, One-way ANOVA with Tukey's post-hoc analysis). S.E. stands for Short Exposure; L.E. stands for Long Exposure. (Corresponds to Fig. 3j).

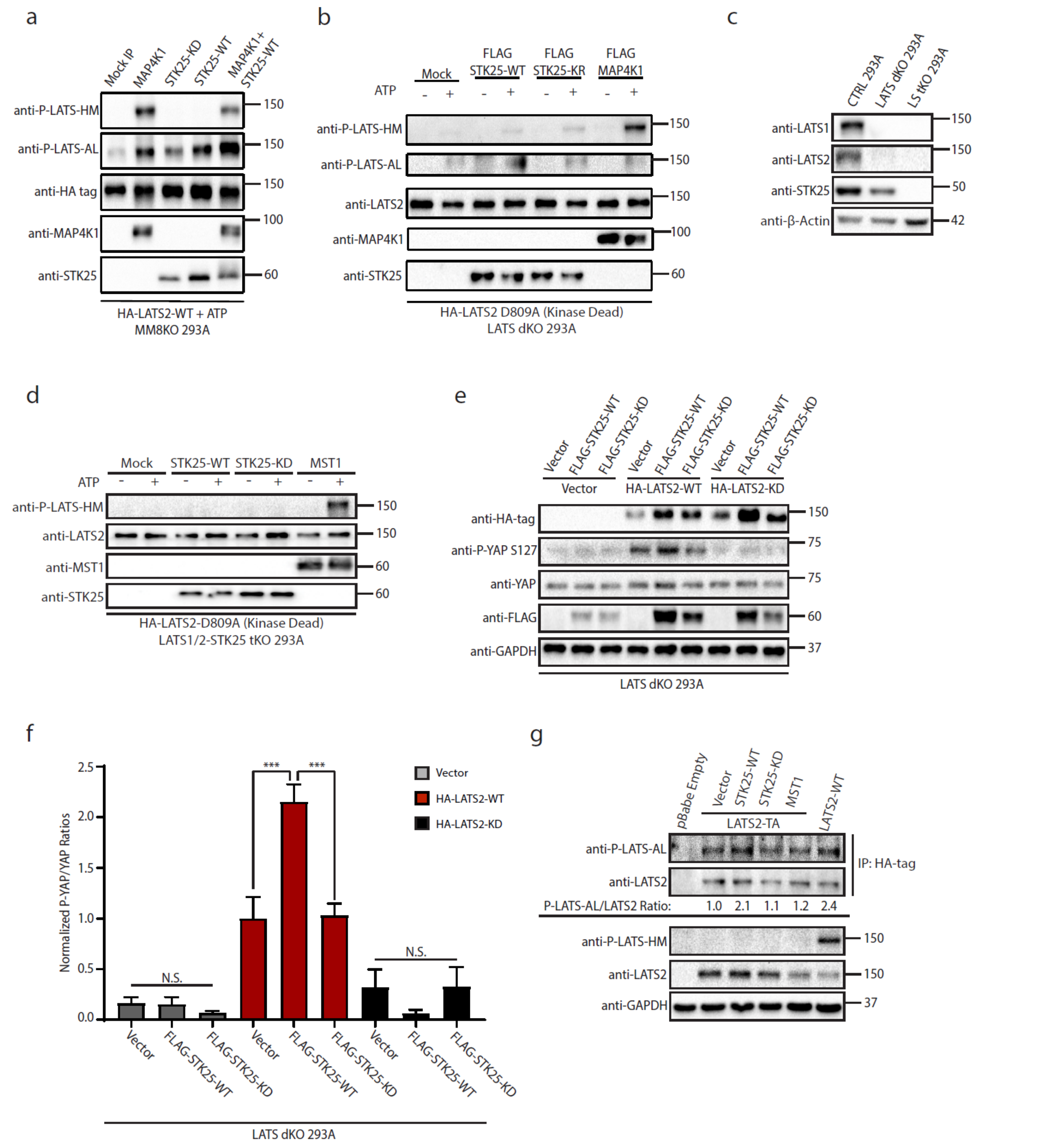

Supplementary Figure 4. STK25 activates the LATS kinases.

a. IP purified wild-type LATS2 (HA-LATS2-WT) from transfected MM8KO 293A cells was co-incubated with IP purified FLAG-STK25-WT, FLAG-STK25-KD, or FLAG-MAP4K1, all from transfected MM8KO 293A. Kinase reactions were allowed to occur in the presence of 500 μM ATP, and levels of phosphorylated LATS at the hydrophobic motif (P-LATS-HM) and activation loop motif (P-LATS-AL) were assessed via immunoblotting. A mock IP product from untransfected MM8KO 293A lysates using FLAG antibody and protein G magnetic beads served as control. **b.** IP purified kinase-dead LATS2 (HA-LATS2-KD) from transfected LATS dKO 293A cells was co-incubated with IP purified FLAG-STK25-WT, FLAG-STK25-KD, or FLAG-MAP4K1, all from

transfected LATS dKO 293A. Levels of phosphorylated LATS at the hydrophobic motif (P-LATS-HM) and activation loop (P-LATS-AL) were assessed via immunoblotting. **c.** Immunoblot to confirm absence of LATS1 and LATS2 in LATS dKO 293A cells, as well as the absence of STK25 in LATS1/2-STK25 triple KO (LS tKO) 293A cells. β-actin served as loading control. **d.** IP-purified kinase-dead LATS2 from transfected LS tKO 293A cells was co-incubated with IP purified FLAG-STK25-WT, FLAG-STK25-KD, or FLAG-MST1, all from transfected LS tKO 293A. Levels of phosphorylated LATS at the hydrophobic motif (P-LATS-HM) was assessed via immunoblotting. **e.** LATS dKO 293A were transfected with empty vector, wild-type LATS2 (HA-LATS2-WT), or kinase-dead LATS2 (HA-LATS2-KD), alongside Vector, wild-type STK25 (FLAG-STK25-WT), or kinase-dead STK25 (FLAG-STK25-KD). Lysates were collected and assessed via SDS-PAGE and immunoblotting for YAP phosphorylation at serine127. **f.** Quantitation of YAP phosphorylation under the various transfection conditions from **e.** are presented (n=3 biological replicates, ***$p<0.001$, One-Way ANOVA with Tukey's post-hoc test; DF=18, F=25.93). **g.** LATS dKO 293A were transfected with HA-tagged hydrophobic motif mutant LATS2 ^{T1041A} (LATS2-TA) alongside either Vector, FLAG-STK25-WT, FLAG-STK25-KD, or FLAG-MST1. HA-tagged LATS2 ^{WT} expression served as positive control. The transfected LATS2 kinases were immunoprecipitated using an anti-HA-tag antibody and assessed by immunoblotting for levels of phosphorylated LATS-AL. All data presented as mean ± SEM.

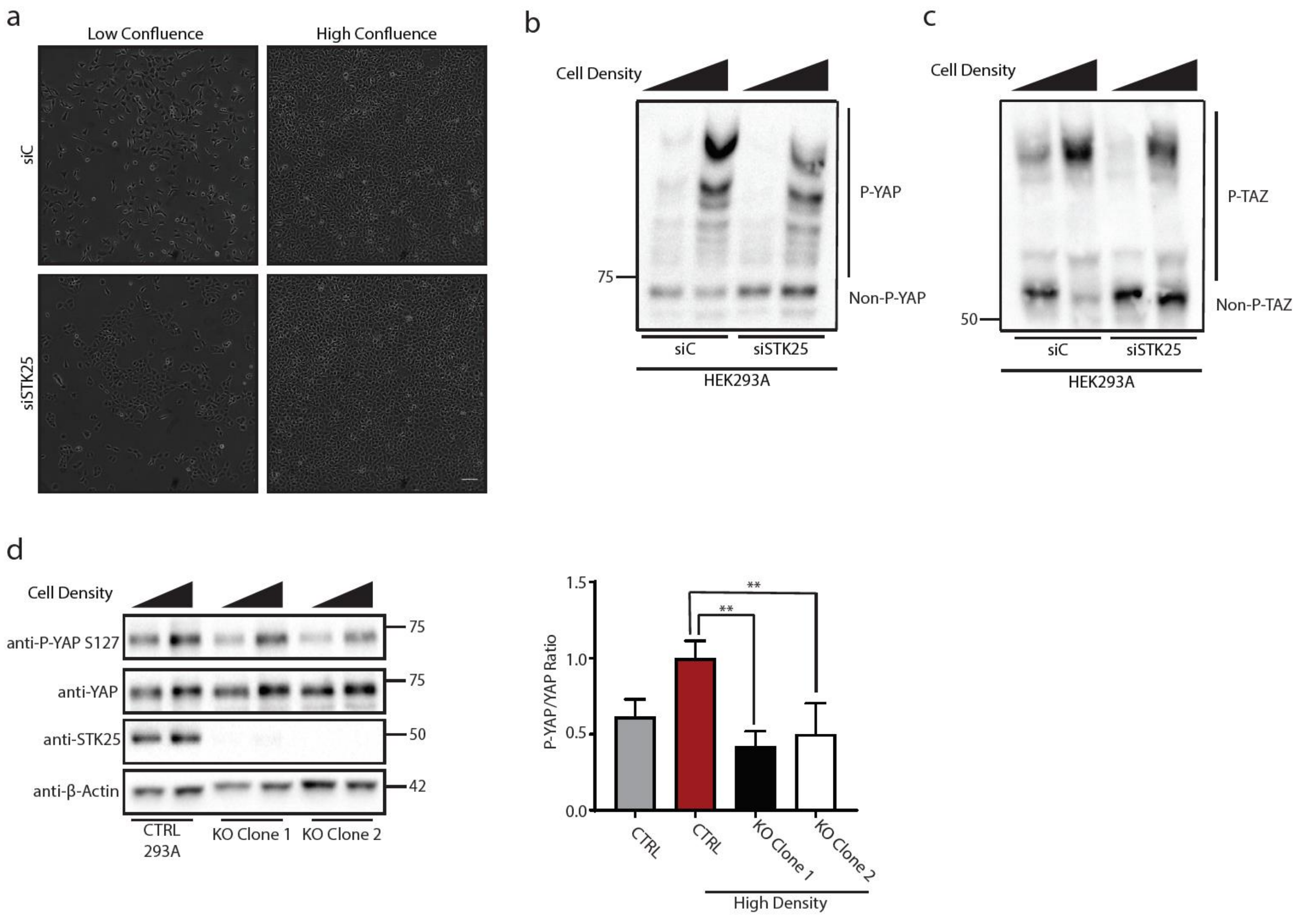

Supplementary Figure 5. STK25 loss impairs physiologic Hippo activation.
a. Representative phase images of HEK293A grown to low or high confluence following transfection with the indicated siRNA. Scale bar, 100 µm. **b.** Global phosphorylation status of YAP was assessed via phos-tag gel electrophoresis using lysates from HEK293A grown to either low or high confluence following transfection with the indicated siRNA. **c.** Global phosphorylation status of TAZ was assessed via phos-tag gel electrophoresis using lysates from HEK293A grown to either low or high confluence following transfection with the indicated siRNA. **d.** Immunoblot and quantitation of phosphorylated YAP levels under conditions of low and high confluence in either control HEK293A stably expressing Cas9 and a non-targeting sgRNA or STK25 KO 293A stably expressing Cas9 together with either sgRNA 1 (Clone 1) or sgRNA 2 (Clone 2) targeting STK25. (n=4 biological replicates; **p<0.01, One-way ANOVA with Dunnett's post-hoc analysis). Data is presented as mean ± SEM.

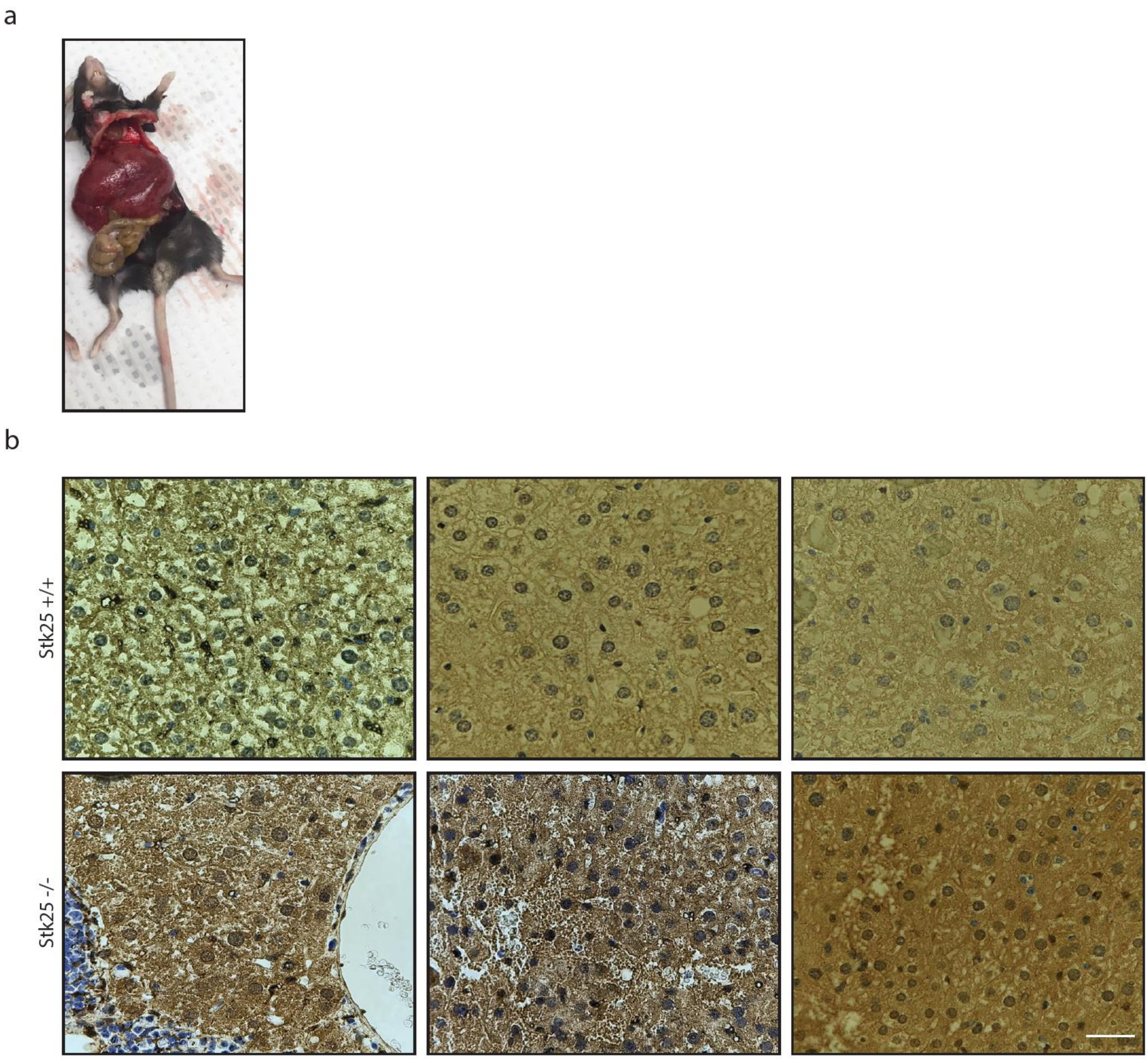

Supplementary Figure 6. Loss of STK25 increases YAP levels *in vivo*.
a. Photographic images of a *STK25-/-* female mouse that spontaneously developed massive hepatomegaly at approximately 11 months of age. **b.** Representative 40X images of IHC staining for YAP in liver sections from *STK25+/+* and *STK25-/-* mice. Each image panel was obtained from a different mouse. Scale bar, 100 µm.

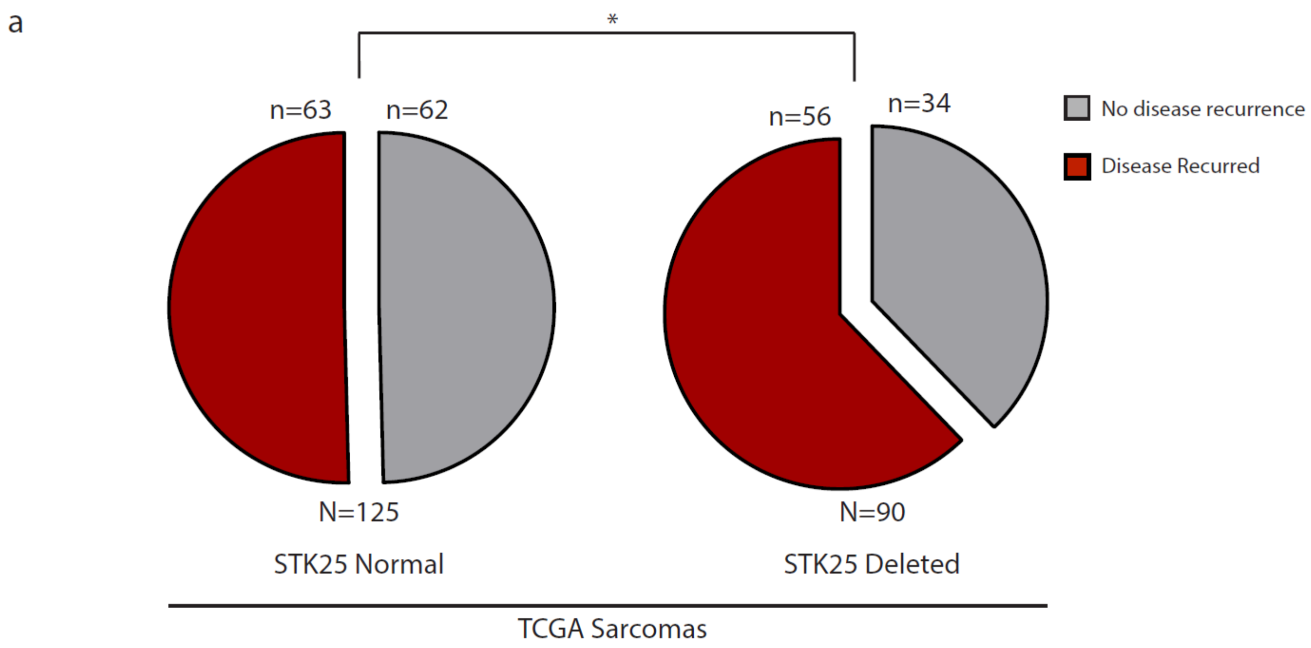

Supplementary Figure 7. Focal deletions of identified Hippo pathway components are rare.
a. Rates of recurrence-free survival in sarcoma patients with and without deletions in *STK25* (n=125 for patients without deletions, n=90 for patients with deletions, *$p<0.05$, two-tailed chi-square goodness-of-fit test).

Fig. 1a

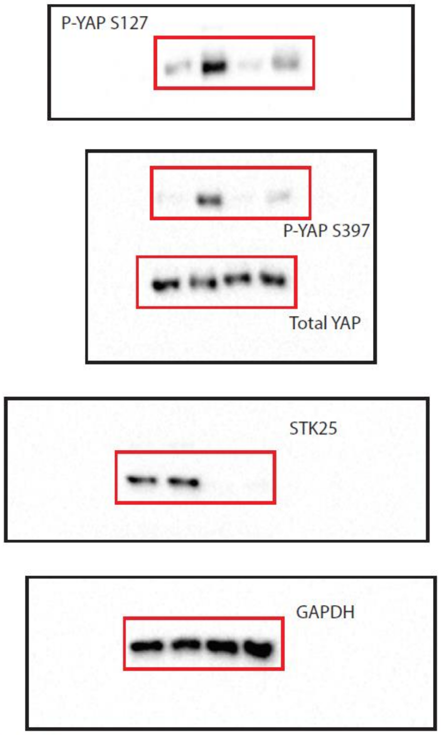

2017-02-23

Fig. 1c

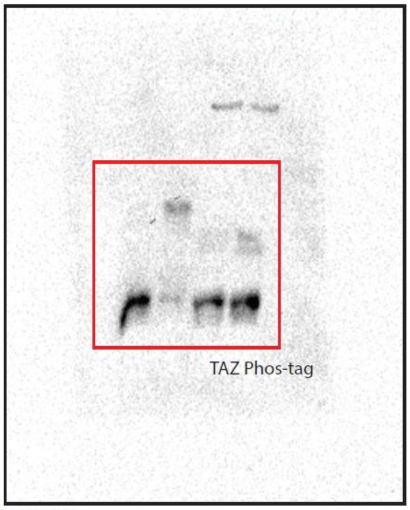

2017-02-17

Fig. 1b

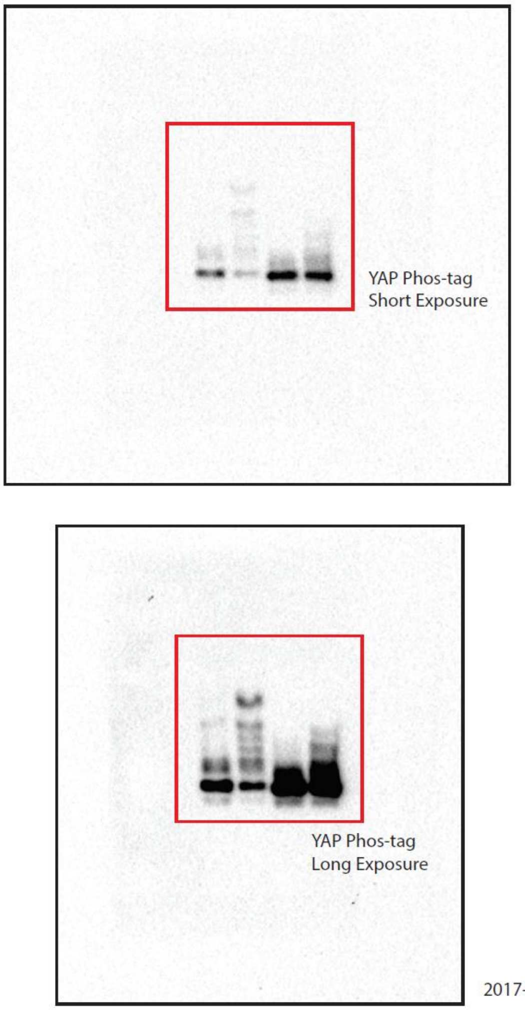

2017-02-17

Fig. 1d

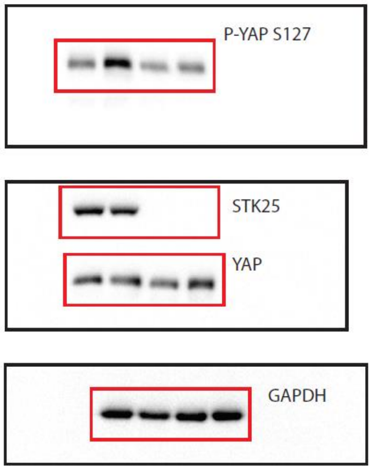

2017-07-03

Fig. 1e

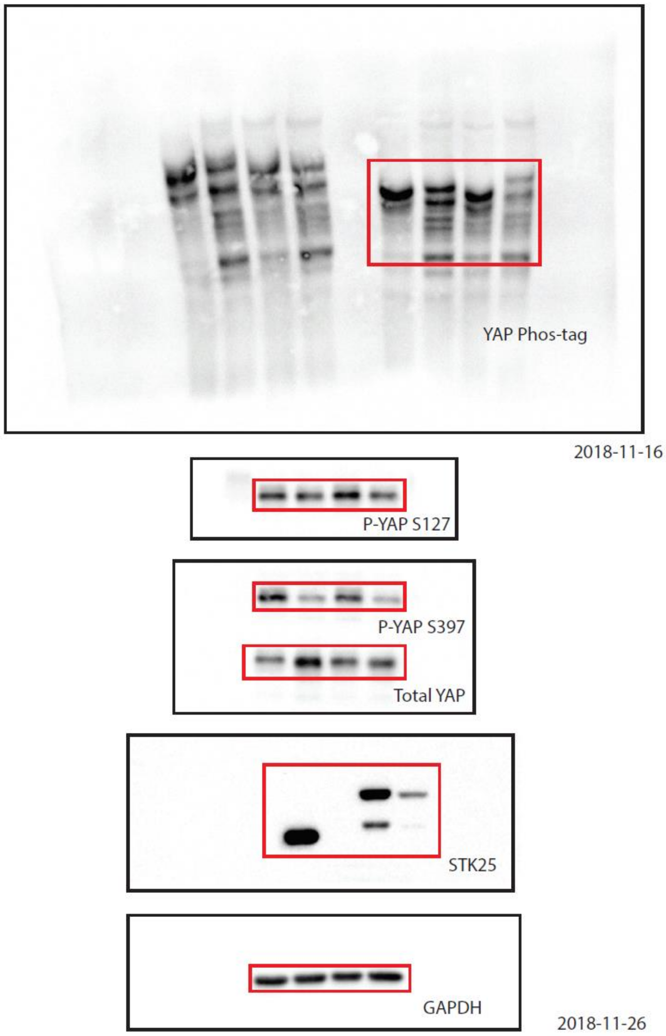

Fig. 3j

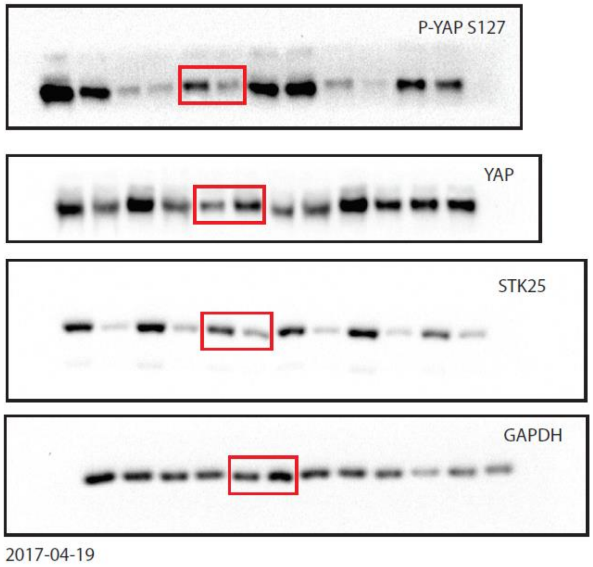

Fig. 3k

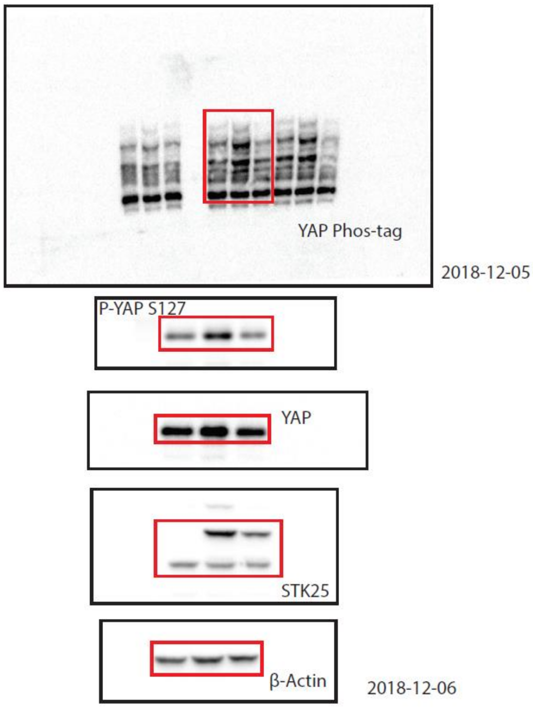

Fig. 4a

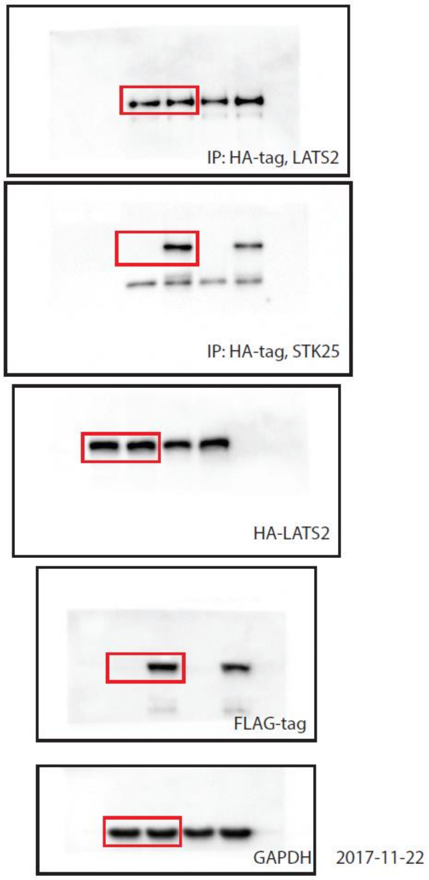

Fig. 4b

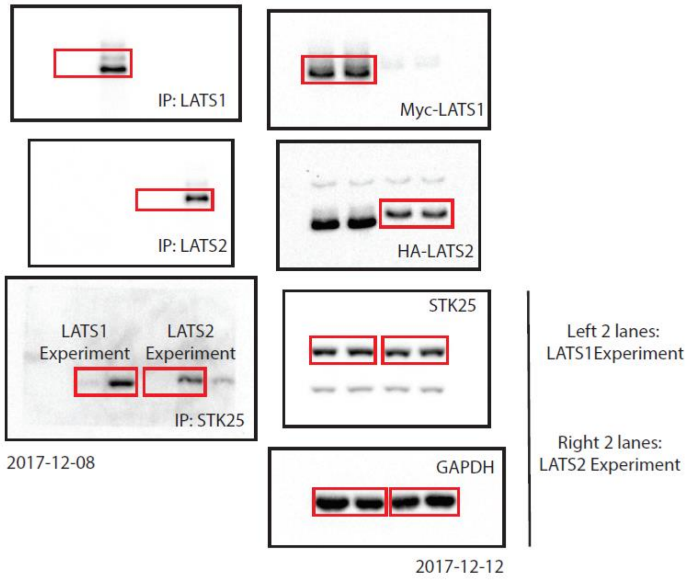

Fig. 4d

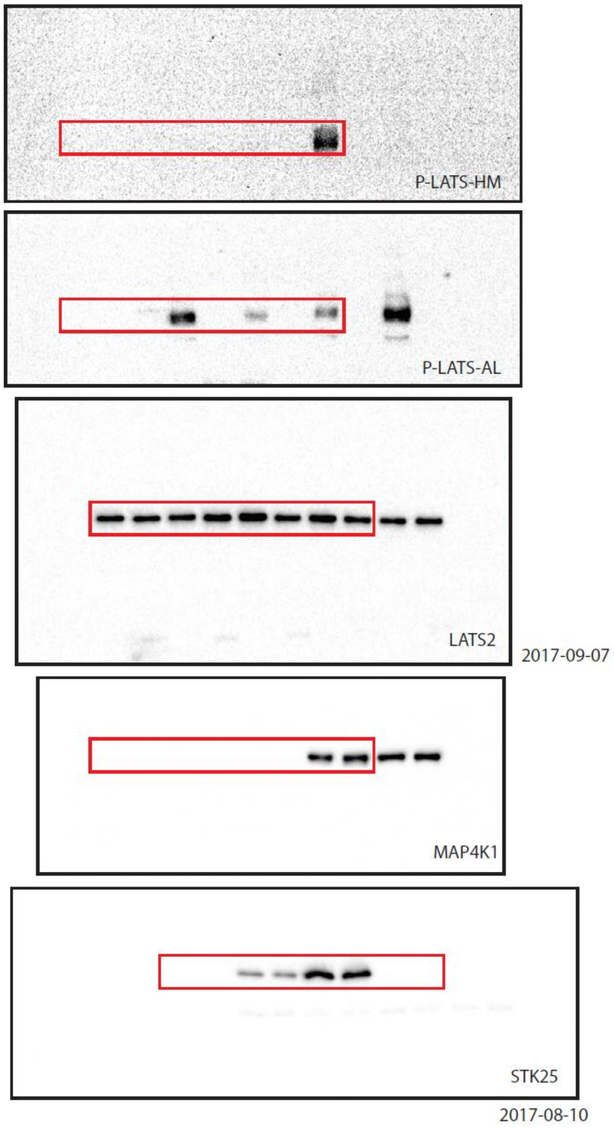

Fig. 4e

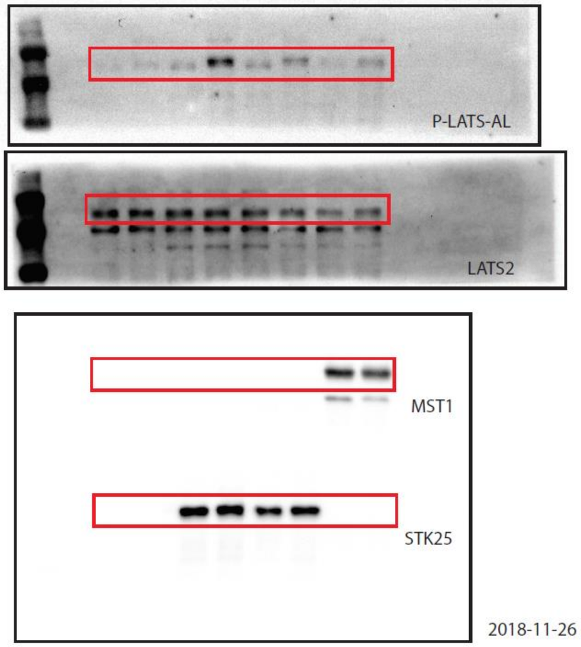

Fig. 4f

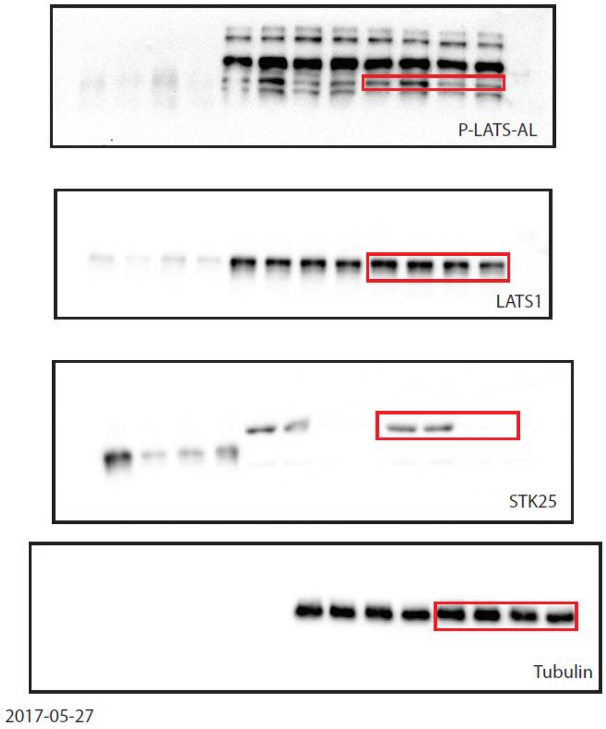

Fig. 4g

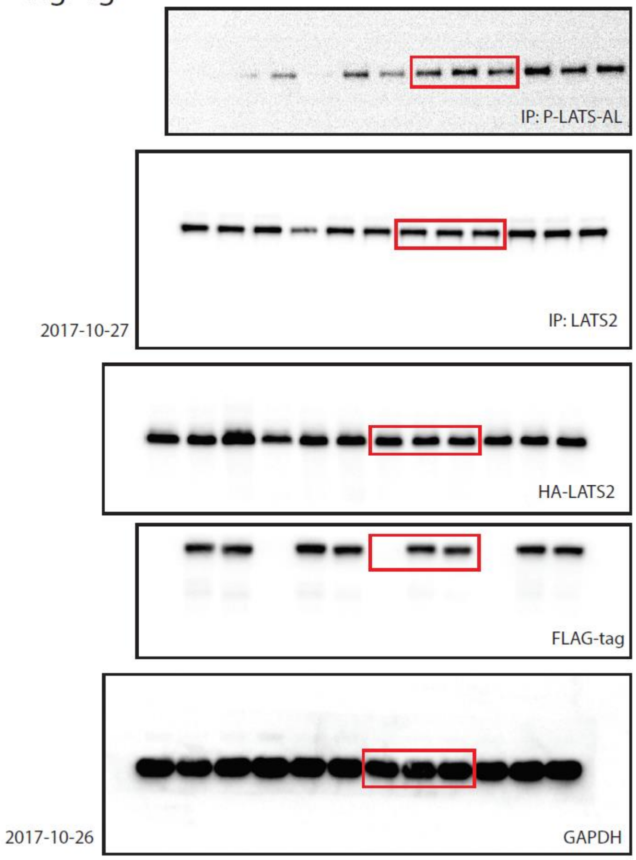

Fig. 4h

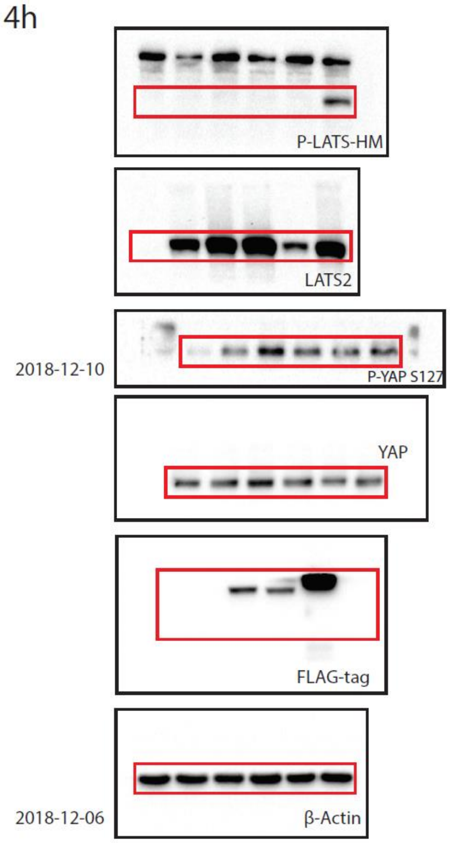

Fig. 5a

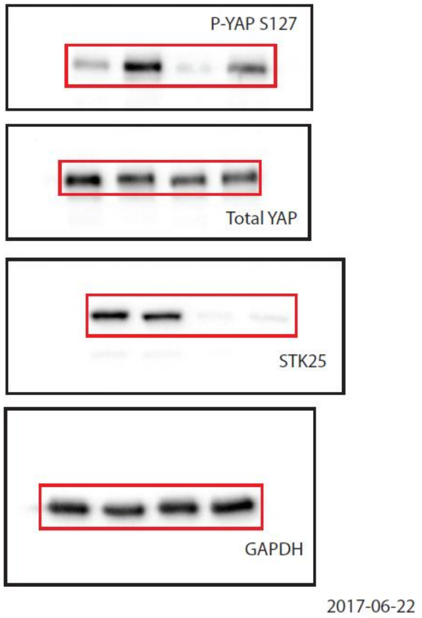

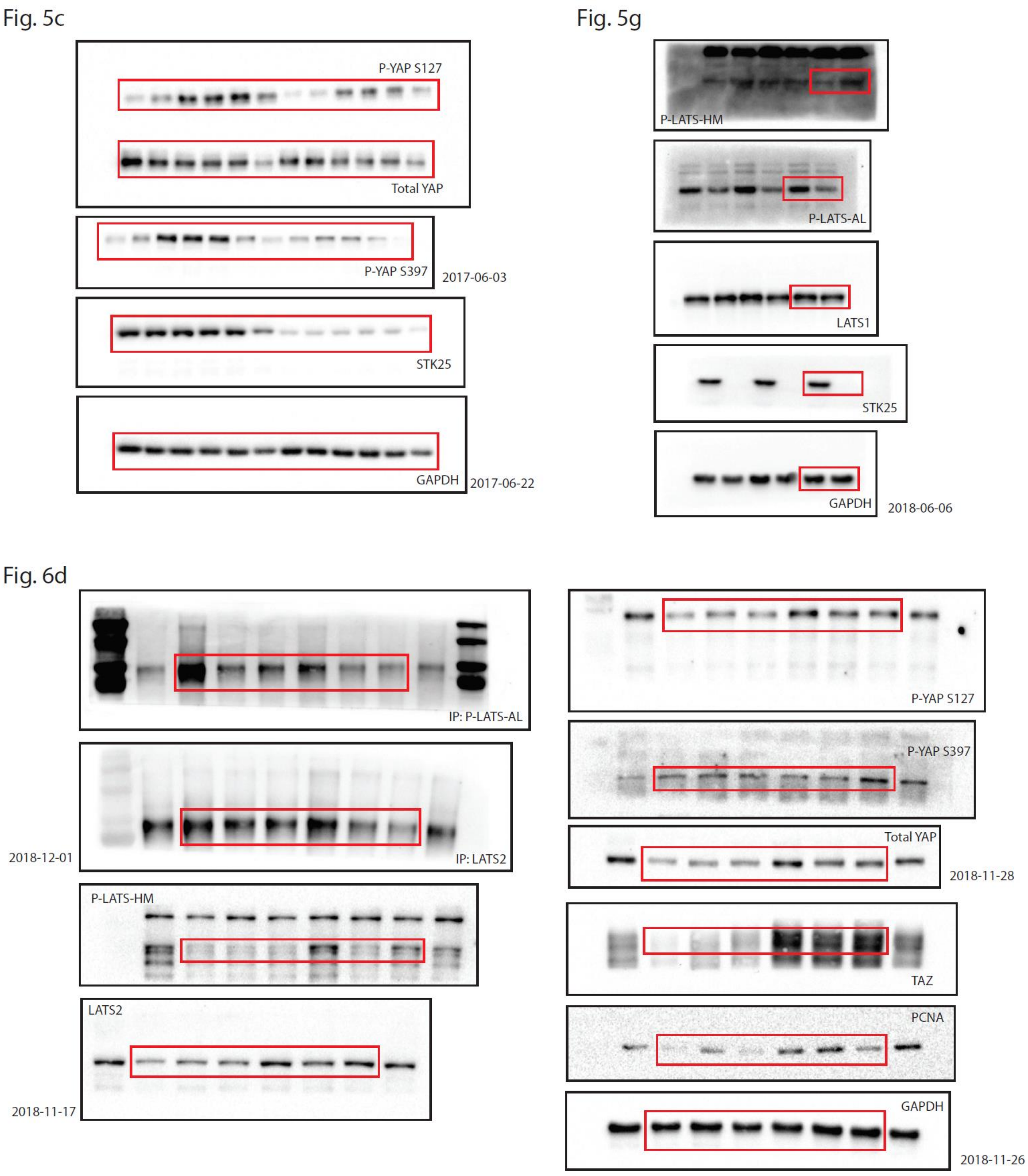

Supplementary Figure 8. Uncropped Original Images of Immunoblots

Uncropped digital originals from which main figure immunoblots were assembled are presented here in linear order. Please note that the auto-scale function on ImageLab was further used to process immunoblots from their original states for final figure assembly.

Annotated Cellular Function/ Published YAP/TAZ gene set	Enrichment Score	Normalized Enrichment Score	FDR q-value
**Park et al. 2016**	**0.62**	**3.17**	**0.000**
HALLMARK_MYC_TARGETS_V1	*0.64*	*3.06*	*0.000*
HALLMARK_E2F_TARGETS	*0.63*	*3.04*	*0.000*
Enzo et al. 2015	***0.59***	***3.01***	***0.000***
**Mohseni et al. 2014**	**0.55**	**2.84**	**0.000**
**Mori et al. 2014**	**0.55**	**2.77**	**0.000**
HALLMARK_OXIDATIVE_PHOSPHORYLATION	*0.56*	*2.66*	*0.000*
**Kim et al. 2016 – YAP/TAZ**	**0.50**	**2.60**	**0.000**
**Zhang et al. 2009 – TAZ**	**0.48**	**2.50**	**0.000**
HALLMARK_G2M_CHECKPOINT	*0.51*	*2.46*	*0.000*

Supplementary Table 1. Loss of STK25 results in enrichment of active YAP/TAZ gene sets. Relative enrichment of active YAP/TAZ gene sets in comparison to enrichment of hallmark gene sets from the Molecular Signatures Database are shown. YAP/TAZ gene sets are in bold, while hallmark gene sets are in italics.

Gene	Amplified/Deleted	Q-value	Overall Frequency
MST1	Neither	N/A	N/A
MST2	Amplified	1.07 E-15	0.4231
MAP4K1	Amplified	1.36 E-37	0.2158
MAP4K2	Neither	N/A	N/A
MAP4K3	Neither	N/A	N/A
MAP4K4	Neither	N/A	N/A
MAP4K5	Neither	N/A	N/A
TNIK/MAP4K6	Amplified	7.75 E-217	0.3284
MINK1/MAP4K7	Neither	N/A	N/A
TAOK1	Amplified	1.55 E-7	0.1857
TAOK2	Neither	N/A	N/A
TAOK3	Neither	N/A	N/A
STK25	Deleted	5.61 E-223	0.1892

Supplementary Table 2. Recurrent focal deletions of upstream LATS activators are rare in cancers. Publicly available TCGA datasets were probed to assess rates of focal deletion in known upstream activators of LATS kinases, using the "All Cancers" dataset from the Tumorscape program online (http://www.broadinstitute.org/tcga/).

Genes in Peak (19)
HDLBP
SEPT2
FARP2
HSA-MIR-3133
**STK25**
BOK-AS1
BOK
THAP4
ATG4B
DTYMK
ING5
D2HGDH
GAL3ST3
NEU4
PDCD1
RTP5
LINC01237
AC093642.3
LOC728323

Supplementary Table 3. List of genes deleted in the *STK25* focal deletion peak.
A list of genes deleted together in the *STK25* focal deletion peak were obtained via TCGA analysis using the "All Cancers" dataset.

Targets	Sequences	Manufacturer
Non-Targeting #1	UGGUUUACAUGUCGACUAA	Dharmacon GE
STK4 SmartPool	CCAGAGCUAUGGUCAGAUA	Dharmacon GE
	GCCCUCAUGUAGUCAAAUA	
	GAUGGGCACUGUUCCGAGUA	
	UAAAGAGACCGGCCAGAUU	
STK3 SmartPool	GCCCAUAUGUUGUAAAGUA	Dharmacon GE
	CCACAAGCACGAUGAGUGA	
	GAACUUUGGUCCGAUGAUU	
	CAUGAACCCUUCCCUAUGU	
STK24 SmartPool	UAUUAUGGAUCCUAUCUGA	Dharmacon GE
	UCGAUUAUCUCCAUUCGGA	
	CCAAGAAUCUCGAGAAUGG	
	AGAAAGUGGUUGCCAUAAA	
STK25 SmartPool	CUAAAGAGCACCAAGCUAU	Dharmacon GE
	UCUACAAGGGCAUCGAUAA	
	ACACGCAGAUUAAGAGGAA	
	GCACUGGACUUGCUUAAAC	
MAP4K1 SmartPool	GAUACAAAUGAGCUGUGUGA	Dharmacon GE
	CAACAACGUUCUCAUGUCU	
	GGAGUUAUCUCUUGGUUGCA	
	GAAAGGACCCUCCAUUGGG	
MAP4K2 SmartPool	GCGCAAAGGUGGCUACAAU	Dharmacon GE
	GGACAGGGACACAAUCCUA	
	GGAAUGACCGCUUGUGGAU	
	CGCCCAAACUGAGAGAUAA	
MAP4K3 SmartPool	CAAUCGAGCUGUUGGAUAA	Dharmacon GE
	GAAGUGUUGUGUUGUAAGA	
	UGUUAACACUGGUGAAUUA	
	GGAGCUAACAUUCUAUUAA	
MAP4K4 SmartPool	GGGAAGGUCUAUCCUCUUA	Dharmacon GE
	GACCAACUCUGGCUUGUUA	
	UAAGUUACGUGUCUACUAU	
	UAUAAGGGUCGACAUGUUA	
MAP4K5 SmartPool	GGAGAGAGAUACCGUUUUA	Dharmacon GE
	CGAAUCAGGUAGUUCAGUU	
	GGUCAUCAACAUUCCAUAA	
	GAACAGUUAUUUCCACGGA	
MINK1 SmartPool	UGAAAUACGAGCGGAUUAA	Dharmacon GE
	UCAUGACUCUGAACCGUAA	
	GGAGGACUGUAUCGCCUAU	
	GAACAGCUAUGACAUCUAC	

TNIK SmartPool	GAACAUACGGGCAAGUUUA	Dharmacon GE
	UAAGCGAGCUCAAAGGUUA	
	CGACAUACCCAGACUGAUA	
	GACCGAAGCUCUUGGUUAC	
LATS1 SmartPool	GGUGAAGUCUGUCUAGCAA	Dharmacon GE
	UAGCAUGGAUUUCAGUAAU	
	GGUAGUUCGUCUAUAUUAU	
	GAAUGGUACUGGACAAACU	
LATS2 SmartPool	GCACGCAUUUUACGAAUUC	Dharmacon GE
	ACACUCACCUCGCCCAAUA	
	AAUCAGAUAUUCCUUGUUG	
	GAAGUGAACCGGCAAAUGC	
TP53 SmartPool	GAAAUUUGCGUGUGGAGUA	Dharmacon GE
	GUGCAGCUGUGGGUUGAUU	
	GCAGUCAGAUCCUAGCGUC	
	GGAGAAUAUUUCACCCUUC	
STK25 siGENOME	GCGCACUGCUGUUCAGAUA	Dharmacon GE
	UAAGAACUGUGCUGACUUG	
	GAAGGUGCCCUGUGCUAUG	
STK25 Flexitube	CCGGCCGAGTCCACACAGCAA	Qiagen
	CACCAAGCTATGGATCATCAT	
	CACGGAGCTCATCGACCGCTA	
	AAGGGCATCGATAACCACACA	
	AAGAACTGTGCTGACTTGGAA	

Supplementary Table 4. List of siRNA sequences used in this study.

Targets	Primer Sequences (5'-3')
**Mouse genes**	
Gapdh Forward	TGTTCCTACCCCCAATGTGT
Gapdh Reverse	GGTCCTCAGTGTAGCCCAAG
Ctgf Forward	CAAGGACCGCACAGCAGTT
Ctgf Reverse	AGAACAGGCGCTCCACTCTG
Cyr61 Forward	TAAGGTCTGCGCTAAACAACTC
Cyr61 Reverse	CAGATCCCTTTCAGAGCGGT
Ankrd1 Forward	AAACGGACGGCACTCCACCG
Ankrd1 Reverse	CGCTGTGCTGAGAAGCTTGTCTCT
Hprt1 Forward	TCAGTCAACGGGGGACATAAA
Hprt1 Reverse	GGGGCTGTACTGCTTAACCAG
Inhba Forward	TCCGAAGGATGGACCTAACTC
Inhba Reverse	GCTTTCTGATCGCGTTGAGAAG
Birc3 Forward	AGAGAGGAGCAGATGGAGCA
Birc3 Reverse	TTTGTTCTTCCGGATTAGTGC
**Human Genes**	
GAPDH Forward	GAGTCAACGGATTTGGTCG
GAPDH Reverse	CATTGATGGCAACAATATCCAC
CTGF Forward	CCAATGACAACGCCTCCTG
CTGF Reverse	TGGTGCAGCCAGAAAGCTC
CYR61 Forward	AGCCTCGCATCCTATACAACC
CYR61 Reverse	TTCTTTCACAAGGCGGCACTC
EDN1 Forward	TGTGTCTACTTCTGCCACCT
EDN1 Reverse	CCCTGAGTTCTTTTCCTGCTT
AMOTL2 Forward	TTGGAATCTGCAAATCGCC
AMOTL2 Reverse	TGCTGTTCGTAGCTCTGAG

Supplementary Table 5. List of qPCR primers used in this study.
