# Supplementary material for: Identification of the kinase STK25 as an upstream activator of LATS signaling
Source: Nat Commun. 2019 Apr 4;10:1547. doi: 10.1038/s41467-019-09597-w (PMC6449379; doi:10.1038/s41467-019-09597-w)
Supplement: Supplementary file 2 — Reporting Summary [file 41467_2019_9597_MOESM2_ESM.pdf]

nature research

Corresponding author(s): Neil Ganem

Last updated by author(s): Feb 27, 2019

Reporting Summary

Nature Research wishes to improve the reproducibility of the work that we publish. This form provides structure for consistency and transparency in reporting. For further information on Nature Research policies, see Authors & Referees and the Editorial Policy Checklist.

Statistics

For all statistical analyses, confirm that the following items are present in the figure legend, table legend, main text, or Methods section.

n/a	Confirmed	
☐	☒	The exact sample size (n) for each experimental group/condition, given as a discrete number and unit of measurement
☐	☒	A statement on whether measurements were taken from distinct samples or whether the same sample was measured repeatedly
☐	☒	The statistical test(s) used AND whether they are one- or two-sided<br>*Only common tests should be described solely by name; describe more complex techniques in the Methods section.*
☒	☐	A description of all covariates tested
☐	☒	A description of any assumptions or corrections, such as tests of normality and adjustment for multiple comparisons
☐	☒	A full description of the statistical parameters including central tendency (e.g. means) or other basic estimates (e.g. regression coefficient) AND variation (e.g. standard deviation) or associated estimates of uncertainty (e.g. confidence intervals)
☐	☒	For null hypothesis testing, the test statistic (e.g. F, t, r) with confidence intervals, effect sizes, degrees of freedom and P value noted<br>*Give P values as exact values whenever suitable.*
☒	☐	For Bayesian analysis, information on the choice of priors and Markov chain Monte Carlo settings
☒	☐	For hierarchical and complex designs, identification of the appropriate level for tests and full reporting of outcomes
☐	☒	Estimates of effect sizes (e.g. Cohen's d, Pearson's r), indicating how they were calculated

Our web collection on statistics for biologists contains articles on many of the points above.

Software and code

Policy information about availability of computer code

Data collection	Imaging Software: NIS Elements-AR 4.13.04, Echo Revolve4 Software. Immunoblot Imaging Software: ImageLab v5.2.1 v11. qRT-PCR data collection: StepOne Software. Luminometric Assays: Gen5 3.00.19.
Data analysis	Graphpad Prism 7.0, Fiji/ImageJ, and Microsoft Excel 2013.

For manuscripts utilizing custom algorithms or software that are central to the research but not yet described in published literature, software must be made available to editors/reviewers. We strongly encourage code deposition in a community repository (e.g. GitHub). See the Nature Research guidelines for submitting code & software for further information.

Data

Policy information about availability of data

All manuscripts must include a data availability statement. This statement should provide the following information, where applicable:

- Accession codes, unique identifiers, or web links for publicly available datasets
- A list of figures that have associated raw data
- A description of any restrictions on data availability

Microarray data deposited under accession codes GSE119502 and GSE119503. TCGA data used in the study are publicly available. The authors declare that all other data supporting this study are available within the article or supplementary figures, or are available from the authors upon reasonable request. Source data pertaining to immunoblots for the main figures (Figs. 1a-e, 3j-k, 4a-b, 4d-h, 5a, 5c, 5g, 6d) can be found in Supplementary Figure 8.

Field-specific reporting

Please select the one below that is the best fit for your research. If you are not sure, read the appropriate sections before making your selection.

☒ Life sciences ☐ Behavioural & social sciences ☐ Ecological, evolutionary & environmental sciences

For a reference copy of the document with all sections, see nature.com/documents/nr-reporting-summary-flat.pdf

Life sciences study design

All studies must disclose on these points even when the disclosure is negative.

Sample size	Sample size was not predetermined for experiments involving cell culture models; a biological triplicate or quadruplicate of experiments were typically performed prior to compilation of data for analysis. For mouse experiments, sample sizes were calculated following power calculations based on similar literature studies.
Data exclusions	No data points were excluded from analysis.
Replication	Experiments were performed using at least three independent biological replicates to ensure reproducibility. All experiments were consistently reproducible.
Randomization	Experimental groups were not randomized. All experiments were performed with appropriate negative controls, as well as positive controls where appropriate.
Blinding	For quantitation of immunofluorescent images, YAP nuclear:cytoplasmic ratios were quantitated in a blinded fashion wherein the identity of the image was hidden to the scientist. Other experiments were not blinded and were performed and analyzed using unbiased methodology.

Reporting for specific materials, systems and methods

We require information from authors about some types of materials, experimental systems and methods used in many studies. Here, indicate whether each material, system or method listed is relevant to your study. If you are not sure if a list item applies to your research, read the appropriate section before selecting a response.

Materials & experimental systems

n/a	Involved in the study	
☐	☒	Antibodies
☐	☒	Eukaryotic cell lines
☒	☐	Palaeontology
☐	☒	Animals and other organisms
☒	☐	Human research participants
☒	☐	Clinical data

Methods

n/a	Involved in the study	
☒	☐	ChIP-seq
☒	☐	Flow cytometry
☒	☐	MRI-based neuroimaging

Antibodies

Antibodies used	Anti-LATS1 (Cell Signaling Technology, 3477), anti-LATS2 (Cell Signaling Technology, 5888), anti-LATS2 (Cell signaling Technology, 13646), anti-LATS2 (Bethyl Labratories, A300-479A), anti-YAP (Cell Signaling Technology, 14074), anti-P-YAP S127 (Cell Signaling Technology, 13008), anti-P-YAP S397 (Cell Signaling Technology, 13619), anti-STK25 (Abcam, ab157188), anti-MST1 (Cell Signaling Technology, 3682), anti-STK3/MST2 (Abcam, ab52641) anti-GAPDH (Cell Signaling Technology, 2118), anti-P-LATS T1079 (Cell Signaling Technology 8654), anti-P-LATS S909 (Cell Signaling Technology, 9157), anti-P-LATS2-S872 (Signalway Antibodies, 12875), anti-PCNA (Cell Signaling Technology, 13110), anti-TAZ (BD Biosciences, 560235), anti-FLAG (Sigma, P2983), anti-MAP4K1 (Abcam, ab33910), anti-β-actin (Cell Signaling Technology, 4970), anti-α-Tubulin (Millipore, CP06-100UG), anti-Myc (ThermoFisher, MA1-33272), horseradish peroxidase-conjugated species-specific secondary antibodies (Cell Signaling Technology, 7074, 7076), species-specific fluorescent secondary antibodies (Molecular Probes, A11001, 11005, A11008)
Validation	All antibodies described above are commercially available antibodies.

Eukaryotic cell lines

Policy information about cell lines

Cell line source(s)	HEK293FT, hTERT-RPE-1, IMR90, HEK293A, STK25 MEFs
Authentication	293FT, hTERT-RPE-1 and IMR90 were purchased directly from ATCC. HEK293A were purchased directly from Invitrogen. STK25 MEFs were obtained from genotype-confirmed E13.5 embryos.

Mycoplasma contamination	All cell lines were regularly passaged into media containing plasmocin (anti-mycoplasmic agent) and thus were not tested for mycoplasma contamination.
Commonly misidentified lines (See ICLAC register)	None of the cell lines used are commonly misidentified.

Animals and other organisms

Policy information about studies involving animals; ARRIVE guidelines recommended for reporting animal research

Laboratory animals	6-12 month old STK25+/+ and STK25-/- C57BL/6 mice were used, taking care to ensure that the overall sex ratios of mice taken from each genotype were equivalent between the two groups.
Wild animals	No wild animals were used.
Field-collected samples	This study did not involve field-collected samples.
Ethics oversight	All animal studies were approved by the WPI Institutional Animal Care and Use Committee.

Note that full information on the approval of the study protocol must also be provided in the manuscript.
